# Prevalence of Total Edentulism and Panoramic Radiographic Findings of Totally Edentulous Patients in a Dental School in Jeddah, Saudi Arabia

**DOI:** 10.7759/cureus.32334

**Published:** 2022-12-08

**Authors:** Mohammed Shammas, Rim Khashfa, Manal Aqlan, Lujain Alahmadi, Hiba Tallah Habib, Elaf Nawawi, Raghad Mona, Razan Sindi

**Affiliations:** 1 Oral and Maxillofacial Rehabilitation/Prosthodontics, Ibn Sina National College for Medical Studies, Jeddah, SAU; 2 Dentistry, Ibn Sina National College for Medical Studies, Jeddah, SAU

**Keywords:** complete edentulous, digital panoramic radiographs, prevalence study, radiographic screening, tooth loss

## Abstract

Background

The prevalence of total edentulism differs significantly between countries and between cities/regions within the same country. It can be affected by various factors, such as age, sex, and socioeconomic factors along with lifestyle and health behavior. Positive findings on panoramic radiographs are frequently observed in totally edentulous patients (TEP) during routine screening.

Objectives

This study aimed to retrospectively determine the prevalence of total edentulism and to evaluate the distribution and location of positive findings on panoramic radiographs of TEP at a dental school in Jeddah, Saudi Arabia.

Methods

A total of 12,428 digital panoramic radiographs of patients aged ≥35 years seeking treatment at a dental school from January 2018 to December 2021 were evaluated to determine the prevalence of total edentulism. Retained root stumps, impacted teeth, radiolucent, and radiopaque lesions were detected on the panoramic radiographs of the TEP. Data were analyzed using descriptive statistics.

Results

Among the 12,428 patients, 521 (4.2%) showed total edentulism on their panoramic radiographs. The prevalence of edentulism was the highest among patients aged >65 years (n=305, 4.5%), men (n=246, 4.2%), and non-Saudi citizens (n=300, 4.4%). A total of 198 radiographic findings were identified in 150 patients. The frequency rates of retained root stumps and impacted teeth were 19.2% (n=100) and 4.6% (n=24), respectively. Radiolucent and radiopaque lesions each accounted for 2.5% (n=13) of the lesions.

Conclusions

The prevalence of edentulism was higher in patients aged >65 years, male patients, and non-Saudi citizens. Retained root stumps were most often encountered among the four positive findings on panoramic radiographs. Considering the high frequency of positive radiographic findings in TEP, panoramic radiographs must be routinely obtained for TEP, but caution must be exercised to reduce the effects of radiation.

## Introduction

Teeth play an indispensable role in the lives of individuals. Regardless of location, country of residence, nationality, race, or color, dental diseases can affect everyone [1-2]. Despite advances in dentistry, tooth loss remains a major public health concern worldwide. The effects of tooth loss, including total edentulism, have been described in several dimensions [2-6]. Total edentulism results in the loss of esthetics, phonation, and mastication as well as social impairment. Therefore, it affects both oral and general health [1,7-10]. Although the prevalence of total edentulism has decreased over the last decade, data from the World Health Organization (WHO) and independent studies in 42 countries show that the prevalence of total edentulism is the lowest in Nigeria (1.3%) and the highest in Bosnia-Herzegovina (78%) among those aged >65 years [1,4,5,11-15]. Few studies have examined the prevalence of total edentulism in Saudi Arabia. Al-Ghannam et al. reported a prevalence of 17% in Al Ahsa [16], Fouda et al., 6% in Dammam [17], Almussallam et al., 1.8% in Riyadh [18], and Mostafa et al., 7.8% in Hail [19]; however, the WHO in 2003 reported it to be 31-46% in the population aged >65 years [1].

As a routine procedure in many dental schools, a thorough clinical examination is supplemented by panoramic radiography when screening new patients [20-22]. Panoramic radiography is a convenient, simple, and rapid method for identifying the general dental health of patients [22]. Although a risk of radiation exposure exists, the benefits outweigh the risks, as numerous studies have shown positive findings in as much as 34.2% of panoramic radiographs [22]. An et al. [21] concluded that the use of panoramic radiography as a supplement to clinical examination is a valuable screening technique. Likewise, positive radiographic findings have been observed in totally edentulous patients (TEP) in many studies conducted worldwide [23-27].

Therefore, data on total edentulism in the population must be obtained, and any changes observed on panoramic radiographs must be recorded periodically. Thus, this study aimed to determine the prevalence of total edentulism, taking into consideration the differences in age, sex, and nationality, and to evaluate the prevalence and location of positive findings on panoramic radiographs of TEP in a dental school in Jeddah, Saudi Arabia.

## Materials and methods

**Figure 1:**
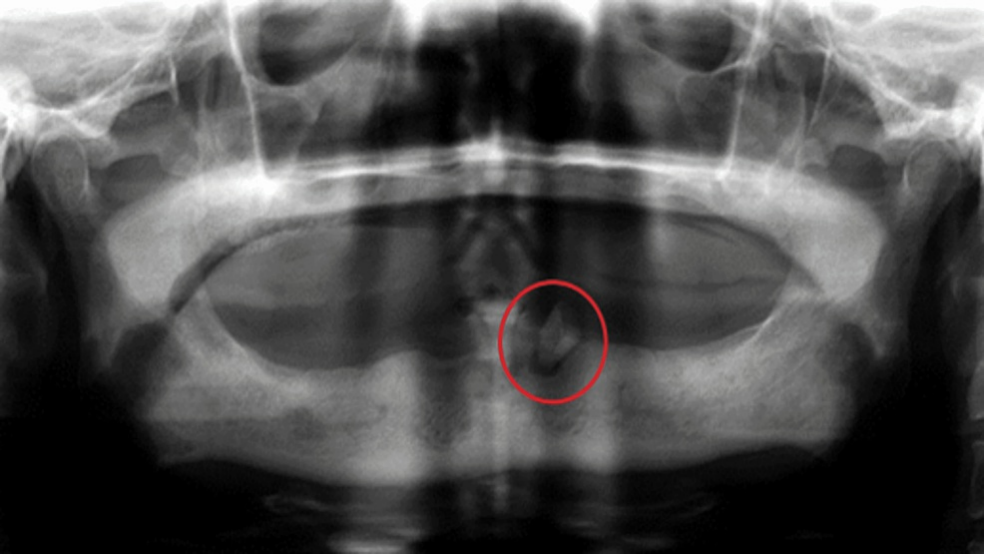
Retained root stump as seen on a panoramic radiograph

**Figure 2:**
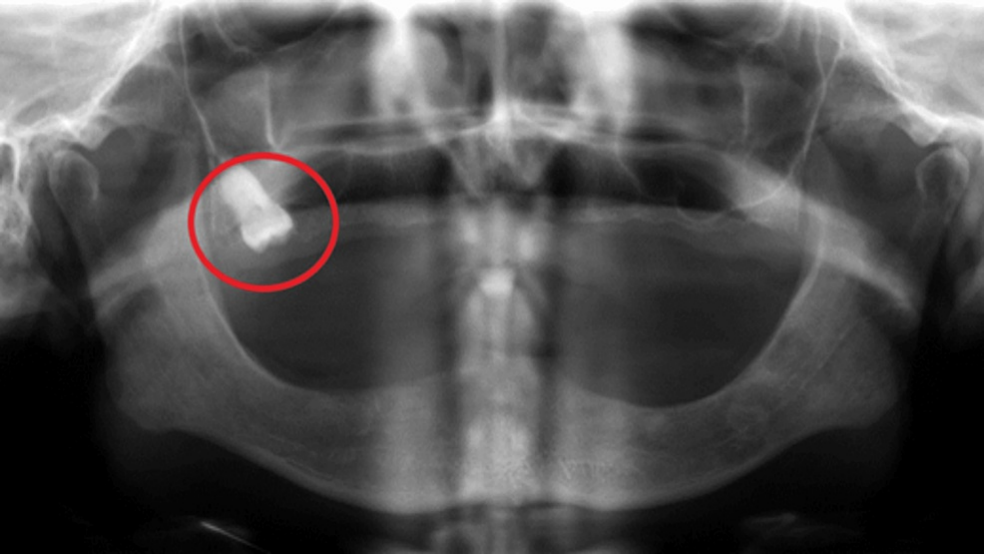
Impacted tooth as seen on a panoramic radiograph

**Figure 3:**
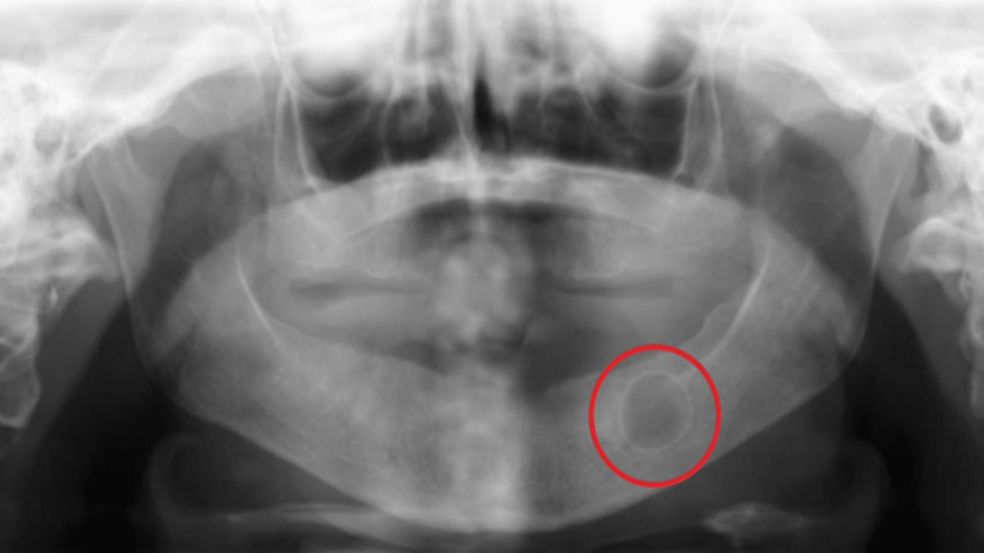
Radiolucent lesion as seen on a panoramic radiograph

**Figure 4:**
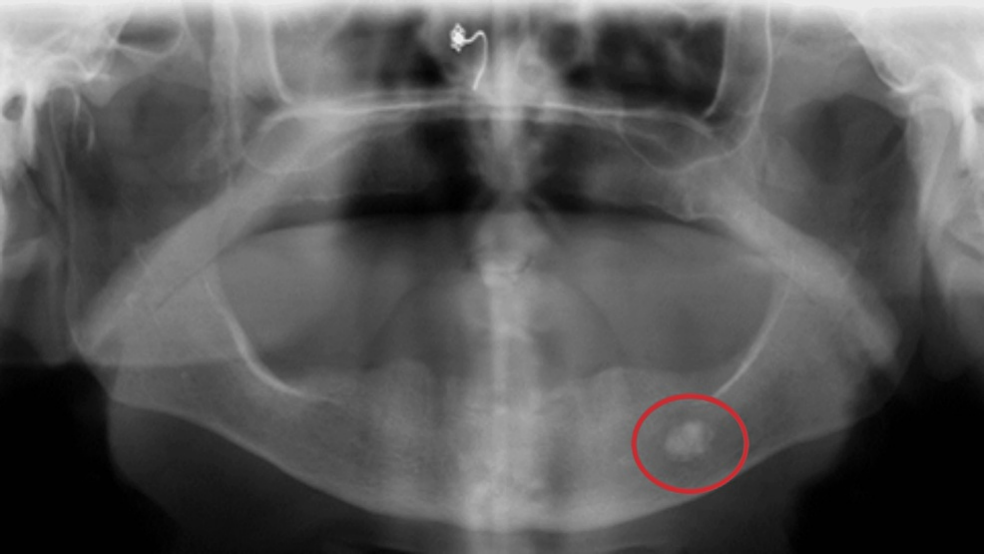
Radiopaque lesion as seen on a panoramic radiograph

This retrospective and descriptive study was conducted in a dental school in Jeddah, Saudi Arabia, after obtaining approval from the Institutional Research Review Board. It included 12,428 digital panoramic radiographs taken from January 2018 to December 2021, which were obtained using a Cranex-D digital cephalometric and panoramic X-ray (Soredx, Tuusula, Finland). The inclusion criteria were age ≥35 years, clear panoramic radiographs, and availability of complete patient data. All patients who sought treatment at our dental school provided informed consent before clinical and radiological examinations. Panoramic radiographs were obtained in accordance with the ethical principles of the Declaration of Helsinki. All radiographs were assessed by a prosthodontist (author MS) and two resident dentists (authors RM and EN). Panoramic radiographs showing no erupted teeth were included in the total edentulism group, and those with at least one tooth were included in the dentulous group. Panoramic radiographs of TEP were evaluated for positive findings, such as retained root stumps, impacted teeth, radiolucent, and radiopaque lesions (Figure 1-4).

Radiographic findings resembling cysts (odontogenic and non-odontogenic) were categorized as radiolucent lesions, and those that resembled chronic inflammation, soft tissue calcifications, fibro-osseous lesions, odontogenic tumors, and bone neoplasms as radiopaque lesions. Foreign bodies were also included in the category of radiopaque lesions. The location of the positive radiographic findings was noted based on the following criteria: the maxilla and mandible were divided into three areas: the right and left posterior areas (the regions of the molar and premolar teeth) and the anterior area (the regions of the incisor and canine teeth). All panoramic radiographs were re-examined to ensure intra-examiner reliability. The findings of some of the panoramic radiographs obtained by one examiner were re-verified by another examiner to improve the inter-examiner reliability. Positive findings on the panoramic radiographs were confirmed by a radiologist. Demographic details such as age, sex, and nationality were also recorded by internship students who participated in this study.

Statistical analysis

All statistical analyses were performed using Microsoft Excel 2016 software program (Microsoft, Redmond, Washington). The obtained data were analyzed using descriptive statistics (counts and percentages). The kappa coefficient was calculated to test intra- and inter-examiner reliability. The kappa coefficient is used to evaluate the agreement between examiners, and a value equal to or greater than 0.81 is considered an "almost perfect agreement".

## Results

Intra- and inter-examiner reliability analyses revealed a kappa coefficient of 0.84 each. Of the 12,428 patients, 6623 (53.3%) were females, 6801 (54.7%) were aged >65 years, and 6836 (55%) were non-Saudi citizens (Indian, Pakistani, Sudani, Egyptian, Yemeni, and Burmese). The mean age of the patients was 50.6 years ± 12.2, with a range of 35 to 87 years. The results are summarized in Table 1.

**Table 1:** Sex-, age-, and nationality-based distribution of the total number of patients from January 2018 to December 2021 (N=12,428) Values are presented as numbers and percentage distribution in brackets

	Total number of patients									
	Males					Females				
	≥35-65 years		>65 years			≥35-65 years		>65 years		
Years	Saudi	Non-Saudi	Saudi	Non-Saudi	Total	Saudi	Non-Saudi	Saudi	Non-Saudi	Total
2018	323 (21.2)	338 (22.2)	361 (23.8)	499 (32.8)	1521 (45.5)	320 (17.6)	481 (26.4)	489 (26.8)	532 (29.2)	1822 (54.5)
2019	369 (21.8)	377 (22.3)	444 (26.2)	502 (29.7)	1692 (47.2)	424 (22.4)	504 (26.6)	450 (23.7)	517 (27.3)	1895 (52.8)
2020	199 (20.2)	272 (27.5)	216 (21.8)	302 (30.5)	989 (49.6)	192 (19.1)	208 (20.7)	239 (23.8)	366 (36.4)	1005 (50.4)
2021	332 (20.7)	421 (26.3)	394 (24.6)	456 (28.4)	1603 (45.7)	380 (20)	487 (25.6)	460 (24.2)	574 (30.2)	1901 (54.3)
Total	1223 (21)	1408 (24.3)	1415 (24.4)	1759 (30.3)	5805 (46.7)	1316 (19.9)	1680 (25.4)	1638 (24.7)	1989 (30)	6623 (53.3)

The prevalence of total edentulism was 4.2% (n=521), as observed on panoramic radiographs. The mean age of this group was 58 years ± 8.2 (38-84 years). The prevalence of total edentulism was higher in males (n=246, 4.2%), those aged >65 years (n=305, 4.5%), and non-Saudi citizens (n=300, 4.4%), as seen in Table 2.

**Table 2:** Sex-, age-, and nationality-based distribution of TEP from January 2018 to December 2021 (n=521; 4,2%) Values are presented as numbers and prevalence distribution in brackets TEP - totally edentulous patients

	Totally edentulous patients									
	Males					Females				
	≥35-65 years		>65 years			≥35-65 years		>65 years		
Year	Saudi	Non-Saudi	Saudi	Non-Saudi	Total	Saudi	Non-Saudi	Saudi	Non-Saudi	Total
2018	11 (3.4)	23 (6.8)	20 (5.5)	30 (6)	84 (5.5)	17 (5.3)	27 (5.6)	23 (4.7)	29 (5.4)	96 (5.2)
2019	15 (4)	22 (5.8)	20 (4.5)	20 (4)	77 (4.5)	11 (2.6)	20 (4)	19 (4.2)	21 (4)	71 (3.7)
2020	6 (3)	7 (2.6)	7 (3.2)	10 (3.3)	30 (3)	6 (3.1)	8 (3.8)	8 (3.3)	12 (3.8)	34 (3.4)
2021	8 (2.4)	12 (2.8)	15 (3.8)	20 (4.4)	55 (3.4)	11 (2.9)	12 (2.5)	24 (5.2)	27 (4.7)	74 (3.9)
Total	40 (3.3)	64 (4.5)	62 (4.4)	80 (4.5)	246 (4.2)	45 (3.4)	67 (4)	74 (4.5)	89 (4.5)	275 (4.1)

In 2020, due to COVID-19, very few patients visited the dental school (1994 patients), of whom 64 (3.2%) had total edentulism.

A total of 198 positive radiographic findings were observed in 150 (28.8%) of 521 TEP (Table 3).

**Table 3:** Location and distribution of panoramic radiographic findings observed in the TEP Values are presented as numbers and percentage distribution in brackets TEP - totally edentulous patients

	Years	Posterior Maxilla (Right)	Anterior Maxilla	Posterior Maxilla (Left)	Posterior Mandibular (Right)	Anterior Mandibular	Posterior Mandibular (Left)	Total
Retained root stumps	2018	6 (15.8)	1 (2.6)	12 (31.6)	6 (15.8)	5 (13.2)	8 (21)	38
	2019	7 (17.5)	3 (7.5)	9 (22.5)	6 (15)	8 (20)	7 (17.5)	40
	2020	6 (18.2)	2 (6.1)	8 (24.2)	6 (18.2)	3 (9.1)	8 (24.2)	33
	2021	6 (16.7)	2 (5.6)	8 (22.2)	7 (19.4)	6 (16.7)	7 (19.4)	36
	Total	25 (17)	8 (5.5)	37 (25.1)	25 (17)	22 (15)	30 (20.4)	147
Impacted teeth	2018	1 (16.7)	-	-	2 (33.3)	-	3 (50)	6
	2019	1 (12.5)	-	-	3 (37.5)	2 (25)	2 (25)	8
	2020	2 (50)	-	-	1 (25)	-	1 (25)	4
	2021	2 (28.6)	-	2 (28.6)	2 (28.6)	-	1 (14.2)	7
	Total	6 (24)	-	2 (8)	8 (32)	2 (8)	7 (28)	25
Radiolucent lesions	2018	2 (40)	-	-	1 (20)	1 (20)	1 (20)	5
	2019	1 (33.3)	-	-	-	1 (33.3)	1 (33.4)	3
	2020	-	-	1 (50)	1 (50)	-	-	2
	2021	1 (33.3)	1 (33.3)	1 (33.4)	-	-	-	3
	Total	4 (30.7)	1 (7.7)	2 (15.4)	2 (15.4)	2 (15.4)	2 (15.4)	13
Radiopaque lesions	2018	2 (25)	-	2 (25)	2 (25)	1 (12.5)	1 (12.5)	8
	2019	1 (50)	-	1 (50)	-	-	-	2
	2020	-	-	-	-	-	-	-
	2021	1 (33.3)	-	-	1 (33.3)	-	1 (33.4)	3
	Total	4 (30.7)	-	3 (23.1)	3 (23.1)	1 (7.7)	2 (15.4)	13

The prevalence rates of retained root stumps (n=100), impacted teeth (n=24), radiolucencies (n=13), and radiopacities (n=13) were 19.2%, 4.6%, 2.5%, and 2.5%, respectively. Retained root stumps were the most common radiographic finding (74.2%) among the four positive ones. One to five retained root stumps were observed in 100 patients. Retained root stumps were mostly observed in the left posterior maxillary area (n=37, 22.5%).

Further, 25 impacted teeth (12.6%) were detected in 24 patients, and one patient had bilaterally impacted third mandibular molars. Eight impacted teeth were identified in the maxilla (32%) and 17 in the mandible (68%). The third molars in both the maxilla and the mandible were the most commonly impacted teeth; 13 radiolucent and 13 radiopaque lesions (6.6%) were identified in 13 patients each. Both types of lesions showed a predilection for the maxilla (n=7, 53.8%). An amalgam tattoo (a radiopaque lesion) was also observed in the maxilla.

## Discussion

According to the WHO, oral and general health are strongly affected by the complete loss of natural teeth (edentulism), caused by a multifactorial process that involves biological and patient-related factors [6,28]. Total edentulism can have a markedly deleterious effect on an individual's well-being [29]. However, studies on the prevalence of total edentulism, which is the need of the hour, are scarce. Dental schools play a crucial role in determining the prevalence of total edentulism and thus updating new data [6,7,13,16,17,19]. This helps various governmental and non-governmental agencies set up a verified database that can be used to plan the prosthetic treatments for this patient population, as many do not have access to dental care. Thus, the importance of this study was that it added to the baseline data on the prevalence of total edentulism among adults and older people in Saudi Arabia, including both Saudi and non-Saudi citizens, using panoramic radiographs.

As of June 2021, the population of Saudi Arabia was 34.1 million, 19.4 million men (56.8%) and 14.7 million women (43.2%), according to the report of the General Authority for Statistics, Saudi Arabia [30]. The prevalence of total edentulism reported by the WHO in 2003 (31-46%) varied widely from the values reported by four individual studies conducted in Saudi Arabia (1.8-17%) [16-19]. In this study, we conducted a retrospective radiographic evaluation of 12,428 patients to determine the prevalence of total edentulism at our dental school in Jeddah, Saudi Arabia. The strength of this study is that it was conducted on a large sample of the population over the past four years. The prevalence of total edentulism among patients aged 35 years and above was 4.2%, which is similar to the 6% reported by Fouda et al. in Dammam, Saudi Arabia, but much lower than the global average [17]. A possible reason for such a low prevalence could be that the study considered only panoramic radiographs of TEP. Many panoramic radiographs (n= 669, 5.4%) revealed a complete breakdown of dentition due to extensive periodontal disease and bone loss requiring complete extraction of teeth; some patients with a few (one to three) teeth remaining needed extractions before the placement of complete dentures, as these teeth could lead to poor prognosis of the dentures. If these two sets of patients had been included among the TEP, the prevalence of total edentulism would have increased significantly.

Various studies have indicated a strong association between sex and age of patients with edentulism [17, 28] and showed that the prevalence of total edentulism is higher in female patients, with a greater impact on their oral health and quality of life than that in male patients [4,13,17,28]. Conversely, the prevalence of total edentulism in this study was marginally higher in men (4.2%) than in women (4.1%), similar to the results of studies conducted elsewhere in Saudi Arabia [16-19].

The Saudi Arabian population is rapidly aging. There are 1.19 million (3.9%) citizens aged >65 years [30]. According to Fouda et al., older people have the highest percentage of total tooth loss [17]. The cumulative effects of dental caries and periodontal disease were observed in patients with increased age; both conditions can deteriorate (if they are not adequately treated or prevented), increasing the risk of tooth loss [16,17]. In our study, the prevalence of total edentulism increased from 3.8% (n=216) in patients aged ≥35-65 years to 4.5% (n=305) in patients aged ≥65 years, which was much lower than that reported in other studies globally [4, 11-14] and locally [17-19].

Although a large expatriate population (n=1.22 million, 36.4%) as of June 2021 resides in Saudi Arabia [30], to our knowledge, no study has examined the prevalence of total edentulism in them. In our study, more non-Saudis (n=6836, 55%) sought treatment at our dental school. Some of the reasons for this could be the availability of free dental treatment to everyone and the presence of a large population of expatriates near the dental school's location. The prevalence of edentulism was also higher (n=300, 4.4%) among non-Saudis than among Saudis (n=221, 4%).

In the second part of the study, the frequency and location of positive findings on panoramic radiographs of TEP were evaluated [13,22]. All panoramic radiographs of TEP were screened for retained root stumps, impacted teeth, radiolucent, and radiopaque lesions. The clinical findings of the TEP included in this analysis were not known, as this was a retrospective study. Previous studies have reported different positive radiographic findings among TEP, with the most common ones being retained root stumps and impacted teeth [13,25,26]. We also found retained root stumps and impacted teeth most frequently in the TEP in our study, thus reinforcing the results of previous studies. The reported frequency of retained root stumps in different populations ranges from 9% to 75%, with an average of 27% [24]. Of the 100 patients, 147 retained root stumps were most frequently encountered in our study, constituting 19.2% of the TEP, similar to the results of previous studies. Most of the retained root stumps were found in the maxillary left posterior area (n=37, 25.1%), as reported in previous studies [13,24,26,27]. This finding may be attributed to incorrect extraction techniques and variations in the root numbers and shapes of maxillary premolars and molars. We emphasize the use of pre-and postoperative periapical radiographs and careful extraction of the maxillary posterior teeth, particularly in the left quadrant. In our study, many patients (n=22, 15%) had retained root stumps in the anterior region of the mandible. Most of them had been endodontically treated and may have been used as abutments for overdentures; however, over time, they lost their coronal tooth structure.

The incidence rates of impacted teeth among TEP ranged from 0.9% to 9% in previous studies [24,26,27]. In our study, the rate was 4.6%, which is well within the range reported in the aforementioned studies. As such, impacted teeth pose no threat unless patients have complaints or if complications are associated with them. In asymptomatic cases, the patients need to be informed about it, and they can be left inside the jaws, and prostheses can be placed over them. Impacted teeth are also important in the preoperative planning of dental implants in edentulous jaws. They need to be removed if they come in the way of implant placement.

In previous studies, the prevalence of radiolucencies and radiopacities in edentulous jaws ranged from 0.9% to 2.5% and 1.5% to 12.1%, respectively [23-27]. Radiolucent and radiopaque lesions were the least commonly observed positive findings in our study, at 2.5% (n=13) each in the TEP, in accordance with the results of previous studies [23-27]. Thus, given the frequency of positive findings on panoramic radiographs were high, preprosthetic evaluation of TEP with panoramic radiographs is highly recommended to ensure the provision of high-quality dental treatment while being cautious of the effects of radiation.

Given that our study included patients who visited a dental school, the results cannot be generalized to the entire population of Jeddah, Saudi Arabia. Further studies with diverse samples from multiple dental schools across Saudi Arabia are required to determine the prevalence of total edentulism in Saudi Arabia. However, the results of this study can facilitate further comparative studies and evaluation of future trends in total edentulism and prosthodontic treatment needs at different dental schools.

Additionally, we did not consider terminal dentition, which would have nearly doubled the prevalence of edentulism. The remaining teeth (one to three), which would eventually require extraction, were excluded from this study, and they also would have added to the tally of the prevalence of total edentulism. Lastly, if single-jaw edentulousness, period of edentulousness, and reasons for tooth loss were examined, the results of this study would have been more robust.

## Conclusions

Total edentulism was observed more frequently in older age groups and in men than in women. More non-Saudi citizens were affected by total edentulism than Saudi citizens were. The prevalence of total edentulism will continue to increase as the population ages unless preventive measures are taken, especially in neighborhoods with low socioeconomic development and areas housing expatriate populations. Improving awareness among all stakeholders and the availability of appropriate services are crucial elements for dealing with total edentulism. Considering the high frequency of positive findings, the importance of taking panoramic radiographs of TEP cannot be overemphasized. Panoramic radiographic examinations of the TEP should be routinely performed while considering the effects of radiation.
